# Factors related to suboptimal recovery of renal function after living donor nephrectomy: a retrospective study

**DOI:** 10.1186/s12882-019-1588-3

**Published:** 2019-11-08

**Authors:** Sho Nishida, Yuji Hidaka, Mariko Toyoda, Kohei Kinoshita, Kosuke Tanaka, Chiaki Kawabata, Satoshi Hamanoue, Akito Inadome, Hiroshi Yokomizo, Asami Takeda, Soichi Uekihara, Shigeyoshi Yamanaga

**Affiliations:** 1 Department of General Surgery, Japanese Red Cross Kumamoto Hospital, 2-1-1 Nagamine-minami, Higashi-ku, Kumamoto, 861-8520 Japan; 2 Department of Urology, Japanese Red Cross Kumamoto Hospital, Kumamoto, Japan; 3 Department of Internal Medicine, Japanese Red Cross Kumamoto Hospital, Kumamoto, Japan; 4 Department of Nephrology, Japanese Red Cross Nagoya Daini Hospital, Myoken-cho, Japan

**Keywords:** Renal function, Living donor, Kidney transplant

## Abstract

**Background:**

The renal function of the remaining kidney in living donors recovers up to 60~70% of pre-donation estimated-glomerular filtration rate (eGFR) by compensatory hypertrophy. However, the degree of this hypertrophy varies from donor to donor and the factors related to it are scarcely known.

**Methods:**

We analyzed 103 living renal transplantations in our institution and divided them into two groups: compensatory hypertrophy group [optimal group, 1-year eGFR ≥60% of pre-donation, *n* = 63] and suboptimal compensatory hypertrophy group (suboptimal group, 1-year eGFR < 60% of pre-donation, *n* = 40). We retrospectively analyzed the factors related to suboptimal compensatory hypertrophy.

**Results:**

Baseline eGFRs were the same in the two groups (optimal versus suboptimal: 82.0 ± 13.1 ml/min/1.73m^2^ versus 83.5 ± 14.8 ml/min/1.73m^2^, *p* = 0.588). Donor age (optimal versus suboptimal: 56.0 ± 10.4 years old versus 60.7 ± 8.7 years old, *p* = 0.018) and uric acid (optimal versus suboptimal: 4.8 ± 1.2 mg/dl versus 5.5 ± 1.3 mg/dl, *p* = 0.007) were significantly higher in the suboptimal group. The rate of pathological chronicity finding on 1-h biopsy (ah≧1 ∩ ct + ci≧1) was much higher in the suboptimal group (optimal versus suboptimal: 6.4% versus 25.0%, *p* = 0.007). After the multivariate analysis, the pathological chronicity finding [odds ratio (OR): 4.8, 95% confidence interval (CI): 1.3–17.8, *p* = 0.021] and uric acid (per 1.0 mg/dl, OR: 1.5, 95% CI: 1.1–2.2, *p* = 0.022) were found to be independent risk factors for suboptimal compensatory hypertrophy.

**Conclusion:**

Chronicity findings on baseline biopsy and higher uric acid were associated with insufficient recovery of the post-donated renal function.

## Background

End-stage renal disease (ESRD) substantially increases the risk of death and cardiovascular disease [1–4]. Renal transplantation is the best treatment option for ESRD [5]. In Japan, due to the shortage of deceased donors, 89.2% of renal transplants are from living donors [6]. To minimize the risk of ESRD after donation, the selection of living donors requires great care [7].

The renal function of the remaining kidney in living donors usually recovers up to 60~70% of baseline function through a compensatory hypertrophy mechanism [8, 9]. However, the degree of this compensatory hypertrophy varies from donor-to-donor. The reason for this between-donor difference is unclear; however, considering the wide range of the health status among living donors, the presence of subtle metabolic syndromes or preclinical renal diseases prior to transplantation are possible, [5] which could affect functional renal recovery after the donation.

Despite meticulous efforts to avoid adverse events for living donors, the 15-year risk of ESRD in donors is 3.5 to 5.3 times higher than that of a matched population [10, 11]. Therefore, accurate estimation of the residual glomerular filtration rate (eGFR) is crucial in order to maintain a donor’s life-long renal function and to prevent cardiovascular events.

We hypothesized that donors’ baseline characteristics and findings on baseline renal biopsy would predict the extent of compensatory hypertrophy after renal donation [5, 10]. Therefore, our aim in this study was to identify the factors related to a suboptimal recovery of renal function in living donors after donation.

## Methods

### Study population

We conducted a retrospective analysis of consecutive 111 cases of living renal transplantations performed at our institution from 2011 to 2016. The donor’s split renal function was calculated by using MAG3 scintigraphy to determine the side of the kidney graft. Living donor nephrectomy was performed using a pure retroperitoneoscopic approach. Of these 111 cases, 8 cases were excluded due to unavailability of baseline biopsies (*n* = 3) and loss to follow-up (*n* = 5). The remaining 103 cases were divided into two groups: the compensatory hypertrophy group [optimal group, with a 1-year eGFR ≥60% of the pre-donation eGFR, *n* = 63] and suboptimal compensatory hypertrophy group (suboptimal group, with a 1-year eGFR < 60% of pre-donation eGFR, *n* = 40). The cut-off eGFR of 60% for classification of suboptimal compensatory hypertrophy was based on a previous study that reported a typical range of post-donation eGFR of 62.5~67% from baseline renal function [8]. We evaluated between-group differences in baseline characteristics and findings through the baseline biopsy obtained during kidney transplantation.

### Definition of the measurements

EGFR was calculated using the following formula for the modified IDMS–MDRD Study equation for Japanese individuals: eGFR (ml/min/1.73 m2) = 194 × (Serum creatinine)^-1.094^ × (Age) ^-0.287^ × 0.739 (if female) [12]. EGFRs were assessed at the initial visit and the annual visit at one year after the donation. Japan Diabetes Society (JDS) HbA1c values were converted into National Glycohemoglobin Standardization Program (NGSP) HbA1c values using the following formula, as recommended by the JDS: NGSP value (%) = 1.02 × JDS value (%) + 0.25%. We diagnosed hypertension as follows using the criteria defined by the Japanese Society of Hypertension (JSH): systolic blood pressure ≥ 140 mmHg or diastolic blood pressure ≥ 90 mmHg [13]. Hyperlipidemia was defined as follows using the criteria recommended by the Japan Atherosclerosis Society; low-density lipoprotein cholesterol (LDL-C) ≥140 mg/dl, high-density lipoprotein cholesterol (HDL-C) ≤40 mg/dl, or triglycerides (TG) ≥150 mg/dl [14]. Salt consumption per day and estimated fractional excretion of sodium in urine were calculated using the formula recommended by the Japanese Society of Hypertension [13]. Hyperuricemia was defined as serum uric acid level ≥ 7.0 mg/dl in men and ≥ 6.0 mg/dl in women [15]. In our study, 14 patients were diagnosed with hyperuricemia before donation, and none of them underwent uric acid treatment.

### Pathological diagnosis

Baseline kidney biopsy was defined as biopsy performed at 1 h after re-perfusion during kidney transplant operation. No other biopsies with different timings or causes (e.g. 1-year protocol biopsy or episode biopsy) were included in this study. Pathological findings were evaluated using the current Banff score [16] of the chronic renal changes (at 1 h) identified in the baseline biopsy specimen, namely: interstitial fibrosis (ci), tubular atrophy (ct), arteriolar hyalinosis (ah), and glomerular atrophy. Based on the percentage of the renal cortical area visible, the ci was classified as minimal (≦5%), mild (6–25%), moderate (26–50%), or severe (≧50%), which corresponded to Banff scores of ci of ci-0, ci-1, ci-2, and ci-3, respectively. Ct was similarly categorized according to Banff scores of ct-0, ct-1, ct-2, and ct-3. Ah was classified as none, mild-to-moderate, moderate-to-severe, or severe, corresponding to a Banff score of ah 0, ah 1, ah 2, and ah 3, respectively. Glomerular atrophy was evaluated as the proportion of atrophic glomeruli to the total number of glomeruli in the specimen. Baseline biopsy data were collected retrospectively from the pathology reports.

### Statistical analysis

Between-group differences were evaluated using Student’s t-test for continuous data, and the chi-squared (χ^2^) test for categorical data. We performed a logistic regression analysis, using a forward selection method for sex, body surface area (BSA), and the characteristics with significant between-group differences. All analyses were performed using SPSS (version 20, IBM, Chicago, Illinois, USA). Two-tailed *p*-values ≤0.05 were considered statistically significant. Values are expressed as mean ± standard deviation, unless otherwise specified.

## Results

### Baseline characteristics

The baseline characteristics are shown in Table 1, with the following variables having a higher value in the suboptimal than optimal group: age (optimal versus suboptimal, 56.0 ± 10.4 years old versus 60.7 ± 8.7 years, *p* = 0.018); HbA1c (5.6 ± 0.3% versus 5.8 ± 0.3%, respectively, *p* = 0.016) and uric acid (4.8 ± 1.2 mg/dl versus 5.5 ± 1.3 mg/dl, respectively, *p* = 0.007). Other variables (hypertension, hyperlipidemia, body mass index, and BSA) were not different between the groups, including baseline eGFR (optimal versus suboptimal. 82.0 ± 13.1 ml/min/1.73m^2^ versus 83.5 ± 14.8 ml/min/1.73m^2^, *p* = 0.588).

**Table 1:** Baseline characteristics of living donors

	Optimal group	Suboptimal group	p-value
	n = 63	n = 40	
Age (years)	56.0 ± 10.4	60.7 ± 8.7	0.018
Male, n (%)	19 (30.2)	18 (45.0)	0.126
Height (cm)	159.8 ± 8.3	160.3 ± 8.7	0.769
Weight (kg)	59.3 ± 10.9	61.5 ± 10.7	0.325
Body mass index (kg/m^2^)	23.2 ± 3.4	23.9 ± 3.3	0.290
Body surface area (m^2^)	1.6 ± 0.2	1.6 ± 0.2	0.399
HbA1c (%)	5.6 ± 0.3	5.8 ± 0.3	0.016
Hypertension, *n* (%)	10 (15.9)	7 (17.5)	0.828
Hyperlipidemia, *n* (%)	10 (15.9)	7 (17.5)	0.828
History of smoking, *n* (%), (*n* = 73)	12(28.0)	6(20.0)	0.441
eGFR (mL/min/1.73 m^2^)	82.0 ± 13.1	83.5 ± 14.8	0.588
Uric acid (mg/dl)	4.8 ± 1.2	5.5 ± 1.3	0.007
Blood nitrogen urea (mg/dl)	13.5 ± 4.7	14.0 ± 3.4	0.582
Side of kidney (right), *n* (%)	5 (8.3)	3 (7.5)	1.0
Urine protein (mg/day), (*n* = 74)	74.1 ± 47.5	78.1 ± 56.9	0.746
24-h creatinine clearance (mg/dl)	113.6 ± 35.2	103.5 ± 23.8	0.089
mGFR via MAG3 scintigraphy (ml/min/1.73 m^2^)	114.2 ± 21.9	111.6 ± 20.0	0.554

*eGFR* estimated glomerular filtration rate: 194 × (serum creatinine) − 1.094 × (age) − 0.287 × 0.739 (if female)

### Histological findings

The chronic histological changes on baseline biopsy are shown in Table 2. In terms of ah, ci, and ct, there were no significant differences between the two groups. The combination of ct and ci score (ct + ci≧1) tended to be higher in the suboptimal group, but this between-group difference was not significant. However, the incidence of having both an ah score and ct + ci score ≧ 1 (ah≧1 ∩ ct + ci≧1) was significantly higher in the suboptimal than the optimal group (optimal versus suboptimal, 6.4% versus 25.0%, p = 0.007). The rate of glomerular atrophy was not significantly different between the two groups (optimal versus suboptimal: 9.1% versus 11.4%, *p* = 0.280).

**Table 2:** Chronic histological changes

	Optimal group	Suboptimal group	p-value
	n = 63	n = 40	
ct0, n (%)	48 (76.2)	23 (57.5)	0.084
1, n (%)	15 (23.8)	16 (40.0)	
2, n (%)	0 (0.0)	1 (2.5)	
ci0, n (%)	56 (88.9)	32 (80.0)	0.281
1, n (%)	7 (11.1)	7 (17.5)	
2, n (%)	0 (0.0)	1 (2.5)	
ah0, n (%)	39 (61.9)	23 (57.5)	0.466
1, n (%)	9 (14.3)	7 (17.5)	
2, n (%)	12 (19.0)	10 (25.0)	
3, n (%)	3 (4.8)	0 (0.0)	
ct + ci0, n (%)	47 (74.6)	22 (55.0)	0.156
1, n (%)	10 (15.9)	11 (27.5)	
2, n (%)	6 (9.5)	6 (15.0)	
3, n (%)	0 (0.0)	0 (0.0)	
4, n (%)	0 (0.0)	1 (2.5)	
Glomerular atrophy rate, %	9.1 ± 10.1	11.4 ± 10.5	0.280
Number of glomeruli per biopsy	20.7 ± 9.4	18.1 ± 7.5	0.123
ct + ci≧1 ∪ ah≧1, n (%)	36 (57.1)	25 (62.5)	0.590
ct + ci≧1 ∩ ah≧1, n (%)	4 (6.4)	10 (25.0)	0.007

*ah* ARTERIAL hyalinosis, *ci* chronic interstitial fibrosis, *ct* chronic tubular atrophy

### Post-donation eGFR

Changes in renal function at 1 year after donation are shown in Table 3. EGFR, HbA1c, blood urea nitrogen, and uric acid are significantly higher in the suboptimal group.

**Table 3:** One-year post-donation results

	Optimal group	Suboptimal group	p-Value
	n = 63	n = 40	
eGFR (mL/min/1.73 m^2^)	55.6 ± 9.4	46.0 ± 8.3	< 0.001
Urine protein (mg/day)	90.5 ± 92.0 (*n* = 62)	94.9 ± 69.2 (*n* = 37)	0.799
Uric acid (mg/dl)	5.9 ± 1.6	6.9 ± 2.0	0.005
Blood nitrogen urea (mg/dl)	15.6 ± 3.6	18.1 ± 3.7	0.001
HbA1c (%)	5.6 ± 0.3	5.8 ± 0.3	0.005
Estimated urine sodium excretion (mg/day)	160.7 ± 26.7 (*n* = 62)	157.0 ± 20.7 (*n* = 37)	0.449
Estimated urine creatinine excretion (mg/day)	1135.8 ± 281.0 (n = 62)	1134.3 ± 254.3 (n = 37)	0.422
Salt consumption per day (g/day)	9.5 ± 1.6 (n = 62)	9.2 ± 1.2 (n = 37)	0.449

*eGFR* estimated glomerular filtration rate: 194 × (serum creatinine) − 1.094 × (age) − 0.287 × 0.739 (if female)

EGFR was about 10 ml/min/1.73m^2^ lower in the suboptimal than optimal group (optimal versus suboptimal, 55.6 ± 9.4 ml/min/1.73m^2^ versus 46.0 ± 8.3 ml/min/1.73m^2^, *p* < 0.001). The following variables were worse (higher) in the suboptimal than optimal group: uric acid (optimal versus suboptimal, 5.9 ± 1.6 mg/dl versus 6.9 ± 2.0 mg/dl, *p* = 0.005) and blood urea nitrogen (15.6 ± 3.6 mg/dl versus 18.1 ± 3.7 mg/dl, respectively, *p* = 0.001). HbA1c was also higher in the suboptimal than optimal group (optimal versus suboptimal, 5.6 ± 0.3% versus 5.8 ± 0.3%, p = 0.005).

**Fig. 1:**
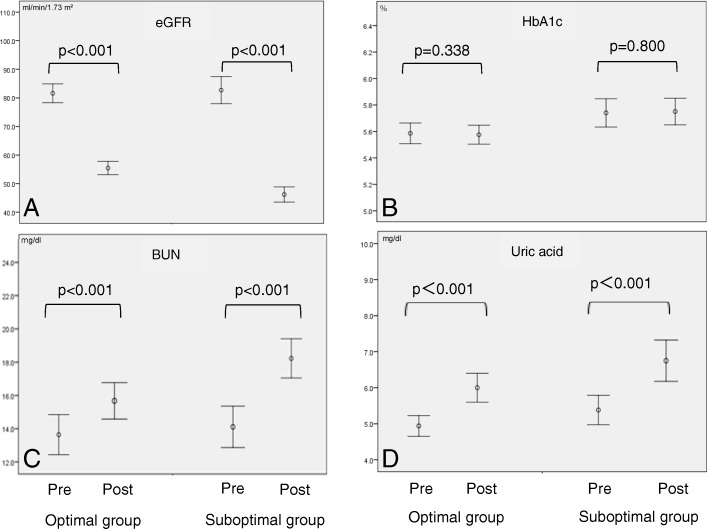
Changes from pre-donation to post-donation of (**a**) eGFR (**b**) HbA1c (**c**) Blood Urea Nitrogen and (**d**) Uric acid

Changes in values from pre-donation to 1-year post-donation are shown in Fig. 1 (A: eGFR, B: HbA1c, C: BUN and D: uric acid). BUN and Uric acid significantly elevated from pre to post in both optimal and suboptimal groups, but not true for HbA1c.

### Multivariate analysis

The logistic regression analyses of the factors related to suboptimal compensatory hypertrophy are shown in Table 4, with the following variables retained as independent predictors: pathological chronicity score (ah≧1 ∩ ct + ci≧1; odds ratio (OR), 4.8, 95%; confidence interval (CI), 1.3–17.8, *p* = 0.021) and uric acid (per 1.0 mg/dl, OR, 1.5; 95% CI, 1.1–2.2, *p* = 0.022).

**Table 4:** Independent risk factors associated with suboptimal compensatory hypertrophy

	Univariate analysis		Multivariate analysis	
	OR (95% CI)	p-value	OR (95% CI)	p-value
ct + ci > 1 ∩ ah ≧1	4.9 (1.4–17.0)	0.012	4.8 (1.3–17.8)	0.021
Age (years, per 10)	1.7 (1.1–2.6)	0.022		
Sex (ref. female)	1.9 (0.8–4.3)	0.128		
Body surface area (m^2^, per 0.1)	2.8 (0.3–3.0)	0.396		
Uric acid (mg/dl, per 1.0)	1.6 (1.1–2.3)	0.010	1.5(1.1–2.2)	0.022
HbA1c (%, per 0.1)	1.2 (1.0–1.3)	0.019		

OR, odds ratio; CI, confidence interval

## Discussion

We identified that hyperuricemia and chronic pathological changes (1 h after biopsy) are independent risk factors for suboptimal compensatory hypertrophy. Although pre-donation eGFRs were not different between the optimal and suboptimal groups, post-donation eGFR was nearly 10 ml/min/1.73m^2^ lower in the suboptimal group in contrast to that in the optimal group.

We defined suboptimal compensatory hypertrophy at 1-year post-donation by an eGFR < 60% from baseline, based on the findings of Colin et al. [8] who reported that renal function after donation recovered to about 62.5~67% of baseline values, which is consistent with the findings in other studies [8, 9, 17, 18]. In addition, the rate of GFR decline was significantly higher in patients with a baseline GFR < 50 ml/min/1.73m^2^ [2, 19, 20]. The risk of cardiovascular events and uremic symptoms significantly increased in patients with an eGFR < 45 ml/min/1.73m^2^, [3, 20] with this risk increasing from 13 to 51%, for an eGFR range of 7.5 to 15 ml/min/m at 1 year [21]. Thus, by setting the cut-off at 60%, we were able to differentiate donors close to chronic kidney disease (CKD) stage IIIA (45~59 ml/min/1.73m^2^) from those with CKD stage IIIB (30~44 ml/min/1.73m^2^), which allowed us to identify the clinically relevant risk factors for suboptimal compensatory hypertrophy.

Interstitial fibrosis and tubular atrophy (IFTA) on baseline biopsy are more closely associated with lower long-term renal function in living donors than other abnormalities, including glomerulosclerosis and arteriolar hyalinosis [10]. However, IFTA is a pattern of injury that has many underlying causes, [22] which is why, in our study, we strived to specify the cause of IFTA by combining ct/ci and ah scores, which identified chronic ischemia induced by arteriosclerosis as the main cause of IFTA. Interestingly, the impact of this combination was independent of age, which is suggestive of a discrepancy between actual and biological age. Moreover, there was no correlation between chronicity score (ah≧1 ∩ ct + ci≧1) and glomerular atrophy. This result was consistent with the well-known fact that tubular atrophy is superior to glomerular pathology as a predictor of declining renal function [23].

It is impractical to obtain a baseline renal biopsy specimen as a component of the primary donor selection process. Instead, Ohashi et al. [5] showed that metabolic syndrome in donors is associated with chronic histological changes in the kidney and subsequent protracted recovery of kidney function after donation. In our study, hypertension, hyperlipidemia, and BMI were not significantly different between the two groups. Furthermore, HbA1c tended to be higher in the suboptimal group, but was not retained as an independent predictor on multivariate analysis. This may be due to the small number of donors. However, uric acid was an independent risk factor for suboptimal recovery of donor renal function. Although the uric acid levels of both groups were in the normal range in our study, this result suggests that higher uric acid levels may be related to the suboptimal recovery of renal function after nephrectomy. Iseki et al. [24] reported a decline in eGFR of 1.91 ~ 4.19 ml/min/1.73m^2^ per 1-mg/dl increment in uric acid, indicative of a role of uric acid in CKD progression. The OR for CKD of 1.4 (95% CI, 1.1~1.8) per 1-mg/dl increment in uric acid, which does not conflict with previous findings by Ficociello et al. [25], who demonstrated a significant association between uric acid and the development of early GFR loss. Sumiyoshi et al. [26] and Nagahama et al. [15] reported that higher uric acid levels were independently associated with a greater risk of incident metabolic syndrome and that hyperuricemia tends to have a clustering of cardiovascular risk factors. In addition, Antonini et al. [27] showed that carotid arterial stiffness is related to uric acid, independently of established cardiovascular risk factors. Although pre-donation hyperuricemia is not included in the donor evaluation guidelines [7], caution should be exerted when hyperuricemia is detected in a donor, regardless of normal renal function.

Some limitations of our study were that it was a single-institution study with a small sample size; further, analysis was retrospective in nature and the follow-up term was relatively short. As biopsies are difficult to perform prior to donor selection, these findings cannot be included in the donor selection process. Additional studies are needed to investigate the added contribution of other factors to the health status of donors, such as pre-sarcopenia, to predict chronic renal pathology from clinical findings.

## Conclusions

Pathological findings in biopsy specimen at 1 h and higher uric acid level were associated with insufficient recovery of renal function at 1 year after donation. Living donors with hyperuricemia and a high chronicity score (ah≧1 ∩ ct + ci≧1) should be followed up with caution after donation.

## Data Availability

The datasets used during the current study are available from the corresponding author on reasonable request.

## Abbreviations

Ah: Arteriolar hyalinosis
BMI: Body mass index
BSA: Body surface area
Ci: Chronic interstitial fibrosis
CI: Confidence interval
Ct: Chronic tubular atrophy
eGFR: Estimated-glomerular filtration rate
ESRD: End-stage renal disease
IFTA: Interstitial fibrosis and tubular atrophy
JDS: Japan Diabetes Society
NGSP: National Glycohemoglobin Standardization Program
